# *Cinnamomum japonicum* Siebold Branch Extracts Attenuate NO and ROS Production via the Inhibition of p38 and JNK Phosphorylation

**DOI:** 10.3390/molecules28041974

**Published:** 2023-02-19

**Authors:** Jae Min Kim, Moon-Hee Choi, Ji Hye Yang

**Affiliations:** 1 College of Pharmacy, Chosun University, Gwangju 61452, Republic of Korea; 2 Department of Biochemical Engineering, College of Engineering, Chosun University, Gwangju 61452, Republic of Korea; 3 College of Korean Medicine, Dongshin University, Naju 58245, Republic of Korea

**Keywords:** *Cinnamomum japonicum* Siebold branches, LPS, macrophage, anti-inflammatory effect

## Abstract

*Cinnamomum japonicum* (CJ) is widely distributed in Asian countries like Korea, China, and Japan. Modern pharmacological studies have demonstrated that it exhibits various biological activities, including antioxidant and anti-inflammatory effects. However, most studies have confirmed the efficacy of its water extract but not that of its other extracts. Therefore, in this study, *Cinnamomum japonicum* Siebold branches (CJB: 70% EtOH extract) were separated using hexane, chloroform, ethyl acetate (CJB3), butanol, and water. Then, their antioxidative activities and phenolic contents were measured. Results revealed that the antioxidant activities and phenolic contents of CJB3 were higher than those of the other extracts. Further, the inhibitory and anti-inflammatory effect of CJB3 on lipopolysaccharide (LPS)-induced reactive oxygen species (ROS) production and LPS-activated macrophages, respectively, was determined. CJB3 suppressed oxidative stress in LPS-activated cells and dose-dependently decreased LPS-stimulated ROS production. CJB3 reduced oxidative stress and reversed the glutathione decrease in LPS-activated RAW264.7 cells. The inhibitory and reducing effect of CJB3 on LPS-induced nitric oxide (NO) production and inducible NO synthase protein and messenger RNA levels, respectively, was investigated. CJB3 inhibited LPS-induced cytokine production and p38 and c-Jun N-terminal kinase (JNK) phosphorylation but not extracellular signal-regulated kinase phosphorylation. Overall, the study results suggest that CJB3 may exert its anti-inflammatory effects via the suppression of p38, JNK, and c-Jun activation.

## 1. Introduction

The production of reactive oxygen species (ROS) is essential for the progression of many inflammatory diseases. ROS can function as intracellular signaling molecules and help regulate diverse biological processes [1,2]. However, excessive ROS production is also important for the elimination of pathogens [3]. Additionally, excessive ROS and insufficient antioxidants induce inflammatory tissue injury [4].

Toll-like receptors (TLRs), mammalian homologs of *Drosophila* Toll, comprise a family of transmembrane proteins that function in immunity and development [5]. TLRs are ubiquitously expressed pattern-recognition receptors that are central to inflammatory responses in various species. It is increasingly demonstrated that a link exists between oxidative stress and TLR signaling [6]. Bacterial lipopolysaccharide (LPS)-induced TLR4 activation triggers ROS expression through multiple mechanisms, including the activation and induction of nicotinamide adenine dinucleotide phosphate (NADPH) oxidase [7] and the suppression of antioxidative enzymes involved in ROS clearance [8]. An intermediate amount of *ROS* triggers an inflammatory response through the activation of nuclear factor kappa-B (NF-κB) and activating protein-1 (AP-1) [9,10], whereas a high oxidative stress level can induce apoptosis [11]. Additionally, LPS triggers ROS production in various cell types, resulting in proinflammatory responses with the expression of various inflammation-associated genes. Therefore, signaling pathways related to inflammatory transcription factors (such as NF-κB and AP-1) and ROS regulation have been key targets of anti-inflammatory drug studies. 

*Cinnamomum japonicum* (CJ) is widely distributed in Asian countries like Korea, China, and Japan. Its bark, commonly known as cinnamon, has a unique fragrance and flavor. Modern pharmacological studies have demonstrated that CJ has various biological activities, including antioxidant, anticancer [12], and anti-inflammatory effects [13]. However, most studies have confirmed the efficacy of CJ water extracts but not that of its other extracts. Therefore, in the present study, *Cinnamomum japonicum* Siebold branches (CJB: 70% EtOH extract) were separated using hexane (CJB1), chloroform (CJB2), ethyl acetate (CJB3), butanol (CJB4), and water (CJB5). All extracts were measured their anti-oxidative activities and phenolic contents. Then, we determined whether CJB3 inhibits LPS-induced ROS production and has anti-inflammatory effect on LPS-activated macrophage. We also identified which transcription factors influenced the inhibition of inflammatory gene expression by CJB3.

## 2. Results

### 2.1. Antioxidative Activities and Phenolic Contents of CJB Extracts

**Figure 1:**
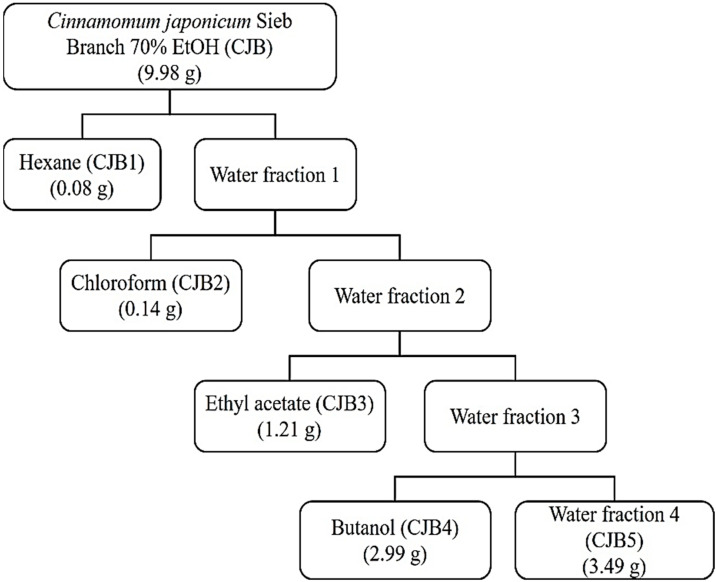
Isolation and fractionation diagram of *Cinnamomum japonicum* Sieb. Branch.

We obtained CJ from Wando-gun, Jeollanam-do, Korea, during the summer of 2020. The fresh CJ branches (CJB) were dried at 40 °C. The dried branches (100 g) were powdered and extraction was conducted (Figure 1).

**Figure 2:**
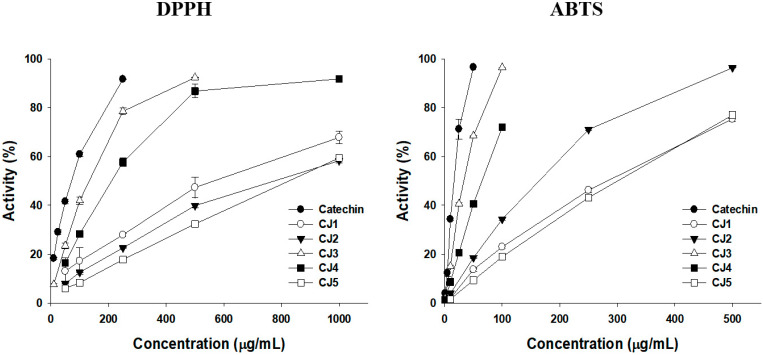
Antioxidant effect of *Cinnamomum japonicum* Sieb. Branch. Antioxidant activity results: 2,2-diphenyl-1-picrylhydrazyl (DPPH) and 2,2-azino-bis (3-ethylbenzthiazoline-6-sulfonic acid) (ABTS).

The antioxidant activities of the fractionated CJB extracts were measured using 2,2-diphenyl-1-picryl-hydrazyl-hydrate (DPPH) and 2,2′-azinobis-(3-ethylbenzothiazoline-6-sulfonic acid (ABTS) radical-scavenging assays. Catechin was used as the positive control. The half-maximal inhibitory concentration (IC_50_) of CJB3 was 144.72 ± 4.58 μg/mL and 35.08 ± 0.06 μg/mL in the DPPH and ABTS radical-scavenging assays, respectively. CJB3 extract exhibited the highest antioxidant activity among the fractionated CJB extracts. In the DPPH radical-scavenging assay, CJB3 exhibited the highest antioxidant activity, followed by CJB4, CJB1, CJB2, and CJB5. In the ABTS radical scavenging assay, CJB3 exhibited the highest antioxidant activity, followed by CJB4, CJB2, CJB1, and CJB5 (Figure 2).

The total polyphenol content (TPC) and total flavonoid content (TFC) were determined using colorimetric methods. Gallic acid and quercetin were used as the equivalent materials. CJB3 had the highest TPC and TFC values of 50.65 ± 1.64 gallic acid equivalent (GAE) mg/g and 143.28 ± 6.34 quercetin equivalent (QUE) mg/g. In the TPC assay, CJB3 exhibited the highest antioxidant activity, followed by CJB4, CJB2, CJB1, and CJB5. In the TFC assay, CJB3 exhibited the highest antioxidant activity, followed by CJB4, CJB2, CJB5, and CJB1. These results suggest that CJB3 had the highest flavonoid and phenolic contents (Figure 2, Table 1).

### 2.2. Identification of CJB3 Components Using HPLC

HPLC analysis was performed to confirm the type of polyphenol contained in CJB3.

**Figure 3:**
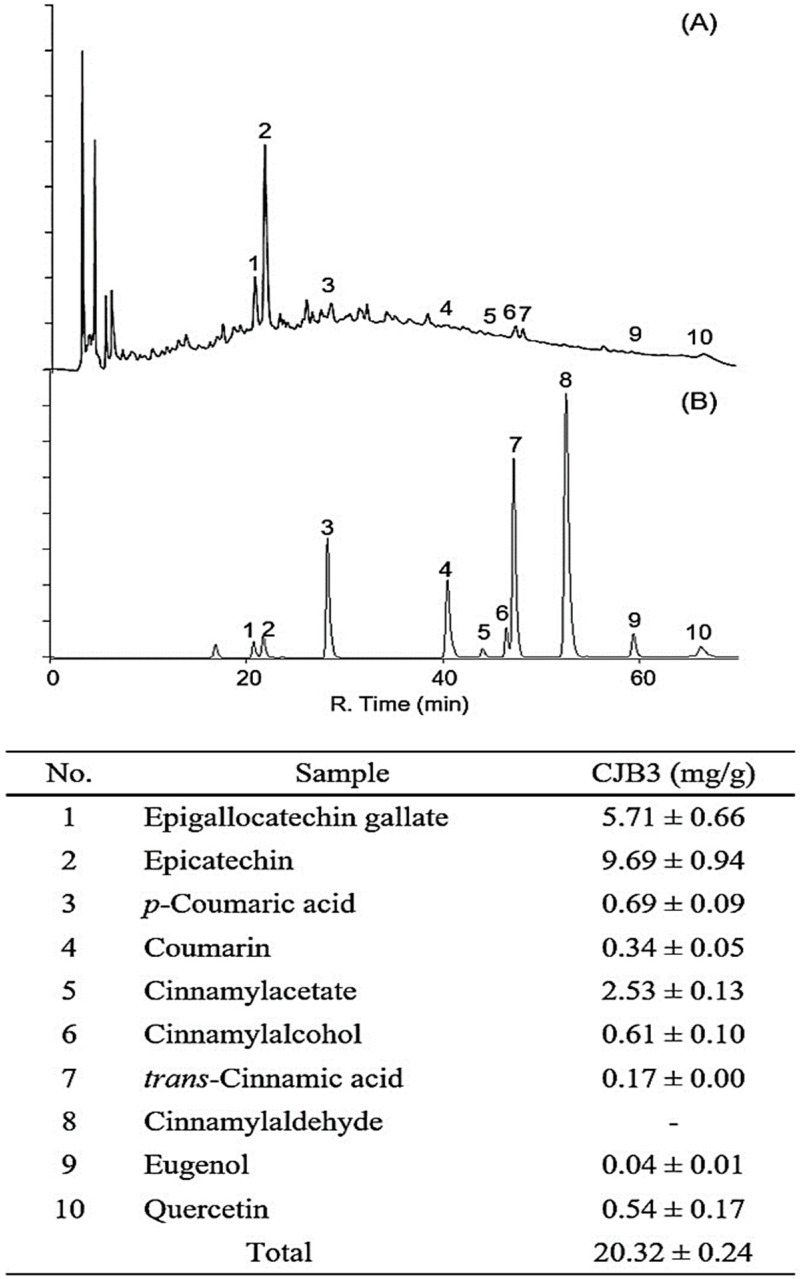
HPLC profile of the CJB3 and standard mixtures using diode array detection at 280 nm. (**A**) Standards and (**B**) CJB3; the numbers indicate the following: (1) Epigallocatechin gallate (2) Epicatechin (3) *p*-Coumaric acid (4) Coumarin (5) Cinnamylacetate (6) Cinnamylalcohol (7) Trans-Cinnamic acid (8) Cinnamylaldehyde (9) Eugenol (10) Quercetin.

Ten phenolic acid and flavonoid standards (epigallocatechin gallate, epicatechin, *ρ*-coumaric acid, coumarin, cinnamyl acetate, cinnamyl alcohol, *trans*-cinnamic acid, cinnamyl aldehyde, eugenol, and quercetin) were selected to identify the CJB3 components (Figure 3). The major component was epicatechin, followed by epigallocatechin gallate and cinnamyl acetate in that order. Cinnamyladehyde was not detected. This is considered to be because cinnamyladehyde is a chloroform-soluble component and is included in CJB2. *Cinnamomum* species have been confirmed to contain large amounts of catechins and proanthocyanidin [14,15,16]. During extraction, the smell of cinnamon was detected. This can be attributed to the presence of cinnamon acetate, which smells like cinnamon, in the leaves and branches of *Cinnamomum* species [17,18]. These research results and our research results are similar, and it was confirmed that flavan-3-ol compounds such as catechins and quercetin were contained. These research results and our research results are similar, and it was confirmed that flavan-3-ol compounds such as catechins and quercetin were contained. LC-MS/MS analysis was performed to confirm HPLC results. We confirmed that quercetin, p-coumaric acid, trans-cinnamic acid, and epigallocatechin gallate contained the most in the order. In particular, Quercetin was identified as having the highest content at 98.82 µg/g (Figure S1).

### 2.3. The Antioxidative Effect of CJB3 on LPS-Activated RAW264.7 Cells

**Figure 4:**
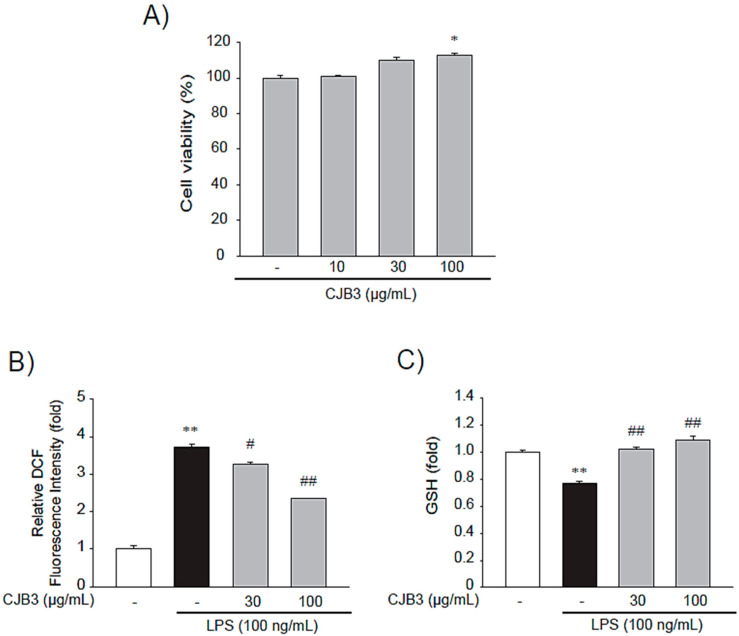
The inhibitory effect of CJB3 on LPS-induced oxidative stress in RAW264.7 cells. (**A**) The cytotoxicity of CJB3 in Raw 264.7 cells: cells were treated with CJB3 (10–100 μg/mL) for 24 h, and cytotoxicity was determined using 3-(4,5-dimethylthiazol-2-yl)-2,5-diphenyl tetrazolium bromide (MTT) assays. The effect of CJB3 on lipopolysaccharide (LPS)-induced reactive oxygen species (ROS) production: cells were treated with CJB3 (30 or 100 μg/mL) for 1 h and incubated with LPS for 3 h. (**B**) Cells were stained with 10 μM 2′-7′-dichlorofluorescin diacetate (DCFH-DA) for 30 min at 37 °C. Intracellular fluorescence intensities were measured using a fluorescence microplate reader. (**C**) The glutathione (GSH) concentrations were measured in the cell lysates treated with LPS and/or 10–30 μg/mL CJB3 for 12 h. Data are expressed as the mean ± standard error of the mean (SE) of three replicates; ** *p* < 0.01, * *p* < 0.05, significant vs. vehicle-treated control; ^##^ *p* < 0.01, ^#^ *p* < 0.05, significant vs. LPS alone.

For the cell experiments, the cytotoxicity of CJB3 in RAW264.7 cells were analyzed first. No cytotoxicity up to 100 μg/mL was observed in the cells (Figure 4A). Next, the suppressing effect of CJB3 on oxidative stress in LPS-activated cells was investigated. ROS-induced oxidative stress plays a major role in inflammatory processes [7]. CJB3 dose-dependently decreased LPS-stimulated ROS production (Figure 4B). CJB3 also inhibited LPS-induced reduced cellular glutathione (GSH) levels (Figure 4C). These results indicate that CJB3 can reduce oxidative stress and recover reduced GSH levels in LPS-activated RAW264.7 cells.

### 2.4. The Suppressing Effect of CJB3 on Nitric Oxide Synthase 

**Figure 5:**
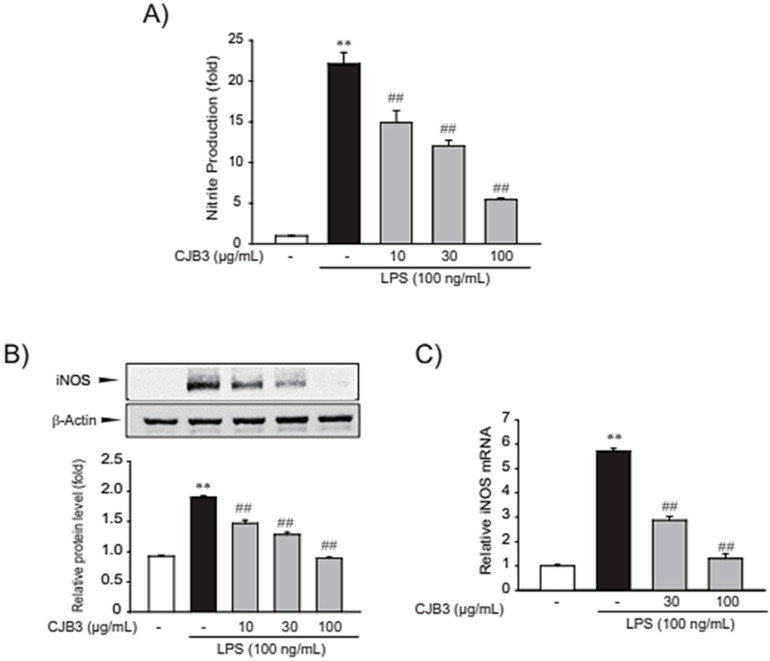
CJB3-mediated inhibition of LPS-induced NO production and iNOS expression. (**A**) Nitric oxide (NO) production: cells were treated with CJB3 (10–100 μg/mL) and/or LPS for 15 h, and NO production was measured using a Griess reagent. (**B**) CJB3-mediated inhibition of inducible NO synthase (iNOS) expression in LPS-activated RAW264.7 cells: cells were pretreated with varying concentrations of CJB3 (10–100 μg/mL) for 1 h and incubated with LPS (100 ng/mL) for 12 h. iNOS protein levels in the cell lysates were measured using western blot. (**C**) The iNOS transcripts were analyzed using RT-PCR assays: cells were pretreated with 30–100 μg/mL CJB3 for 1 h and incubated with 100 ng/mL LPS for 6 h. Data are expressed as the mean ± SE of three replicates; ** *p* < 0.01., significant vs. vehicle-treated control; ^##^ *p* < 0.01, significant vs. LPS alone.

Nitric oxide (NO) has been established as an inflammatory mediator, and inducible NO synthase (iNOS) is expressed predominantly by microglia and macrophages via danger or foreign signal sensing [19]. The anti-inflammatory effect of CSL3 was investigated by determining the level of NO production and iNOS expression in RAW264.7 cells. CJB3 inhibited LPS-induced NO production (Figure 5A) and decreased the expression of iNOS, a protein associated with NO production (Figure 5B). Next, the inhibitory effect of CJB3 on the transcriptional regulation of iNOS in macrophages was investigated. Similarly, CJB3 inhibited LPS-induced messenger RNA (mRNA) levels of iNOS in the cells (Figure 5C).

### 2.5. Effect of CJB3 on LPS-Mediated Production of Proinflammatory Cytokines

**Figure 6:**
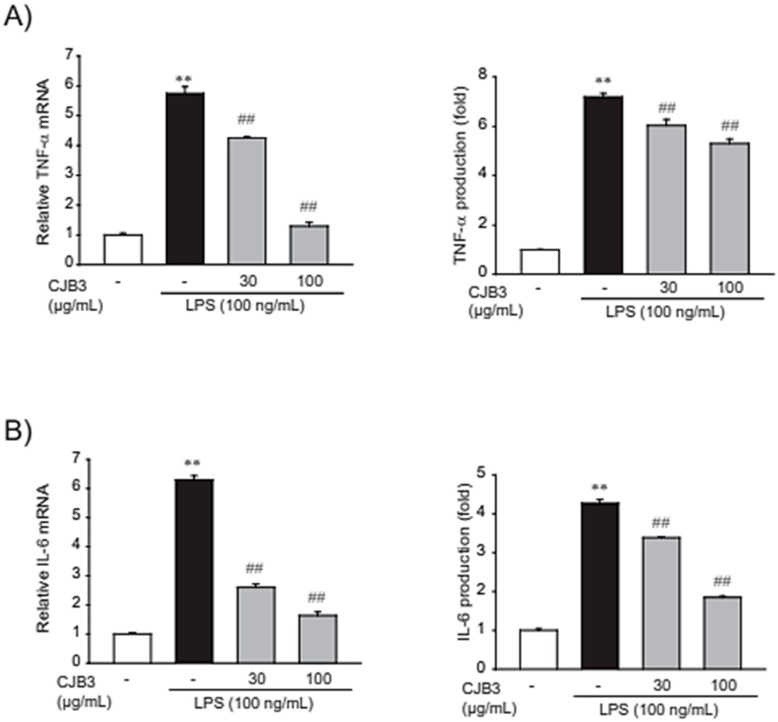
CJB3-mediated inhibition of LPS-induced proinflammatory cytokine expression. (**A**,**B**) Measurement of the inhibitory effect of CJB3 on proinflammatory cytokine expression: cells were treated with 30 or 100 μg/mL CJB3 for 1 h and incubated with LPS for 6 h. (**A**) Tumor necrosis factor-alpha (TNF-α) and interleukin-6 (IL-6) transcripts were monitored using RT-PCR assays. (**B**) Enzyme-linked immunosorbent assay (ELISA): TNF-α and IL-6 release into the culture supernatant was determined using ELISA. Data are expressed as the mean ± SE of three replicates; ** *p* < 0.01, significant vs. vehicle-treated control; ^##^ *p* < 0.01, significant vs. LPS alone.

The effect of CJB3 on proinflammatory cytokines was investigated. Proinflammatory cytokines like tumor necrosis factor-alpha (TNF-α) and interleukin-6 (IL-6) play important roles in immune responses [20]. LPS treatment elevated TNF-α and IL-6 mRNA levels. However, pretreatment with CJB3 significantly counteracted these effects (Figure 6A). CJB3 also inhibited the LPS-induced release of cytokines (TNF-α and IL-6) into the media (Figure 6B). These results indicate that CJB3 alleviates LPS-mediated production of proinflammatory cytokines. 

### 2.6. CJB3-Mediated Inhibition of LPS-Induced Phosphorylation of p38, JNK and c-Jun 

**Figure 7:**
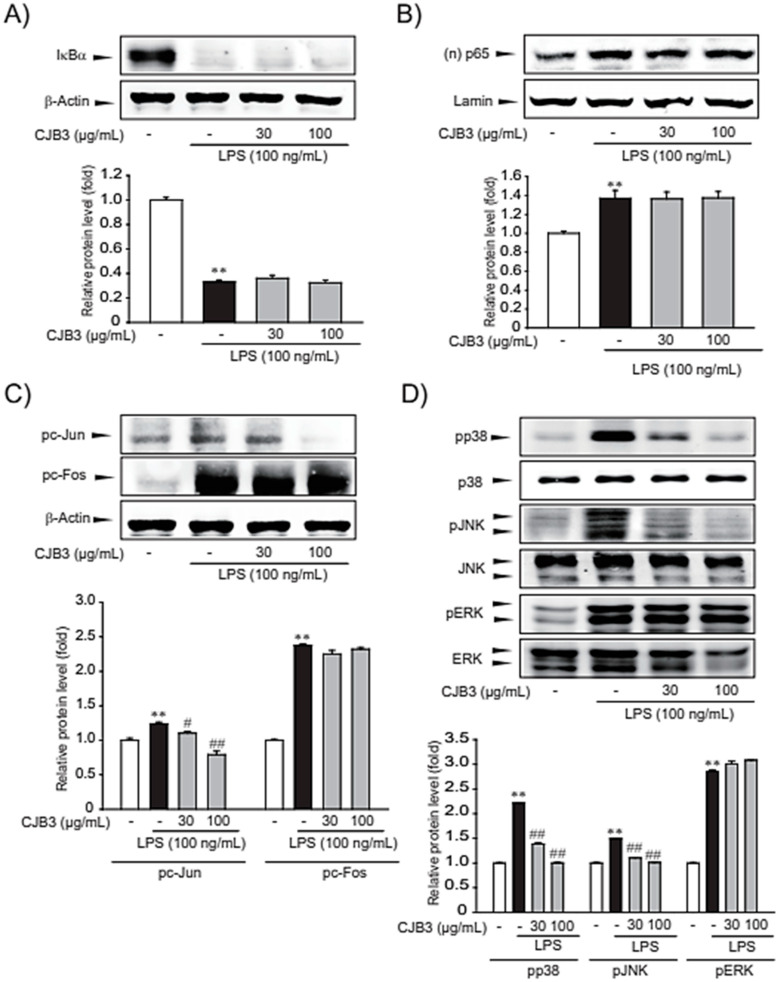
CJB3-induced specific inhibition of c-Jun, p38 and JNK phosphorylation in LPS-activated RAW264.7 cells. (**A**) Immunoblotting for total IκBα: cells were pretreated with CJB3 for 1 h before LPS stimulation for 15 min. Total IκBα in the cell lysate was immunoblotted. (**B**) The expression level of p65 protein in cells with nuclear fraction. Using Lamin as control for nuclear fraction. RAW264.7 cells were pretreated CJB3 for 1 h and then incubated with LPS for 3 h. (**C**) Immunoblotting for c-Jun and c-Fos phosphorylation: cells were pretreated with CJB3 for 1 h before LPS stimulation for 2 h, and cell lysates were immunoblotted to examine c-Jun and c-Fos phosphorylation. (**D**) Effect of CJB3 on LPS-induced phosphorylations of MAPKs: cells were treated with CJB3 for 1 h before LPS stimulation for 30 min. Data are expressed as the mean ± SE of three replicates; ** *p* < 0.01, significant vs. vehicle-treated control; ^#^ *p* < 0.05, ^##^ *p* < 0.01, significant vs. LPS alone.

NF-κB and AP-1 are transcription factors that play crucial roles in inflammation, immunity, cell proliferation, and apoptosis. NF-κB activation primarily occurs via IκB kinase (IKK)-mediated phosphorylation of inhibitory molecules, including IκBα [21]. AP-1, comprised of *c-Fos* and *c-Jun* proteins, is a regulator of cytokine expression and an important modulator of inflammatory diseases like rheumatoid arthritis, psoriasis, and psoriatic arthritis [22]. Therefore, the inhibitory effect of CJB3 on LPS-induced IκBα degradation/NF-κB p65 nuclear translocation and *c-Fos*/*c-Jun* phosphorylation in RAW264.7 cells was investigated first. CJB3 treatment cannot decreae IκBα degradation, p65 (one of the five components that form the NF-κB transcription factor family) nuclear translocation, and *c-Fos* phosphorylation, whereas CJB3 inhibited *c-Jun* phosphorylation (Figure 7A–C). 

Next, the effect of CJB3 on LPS-induced phosphorylation of mitogen-activated protein kinases (MAPKs) was assessed. In macrophages, MAPKs, including extracellular signal-regulated kinase (ERK), p38, and c-Jun N-terminal kinase (JNK), are crucial mediators of proinflammatory cytokine production. The assessment results revealed that CJB3 inhibited LPS-induced phosphorylation of p38 and JNK but not ERK (Figure 7D). Overall, the study results suggest that the anti-inflammatory effect of CJB3 might involve the suppression of p38, JNK, and c-Jun activation. 

## 3. Discussion 

Modern pharmacological studies have demonstrated that CJ exhibits various biological activities, including antioxidant and anti-inflammatory effects. However, most studies confirmed that the efficacy of *Cinnamomum japonicum* water extracts, but not other extracts. Antioxidant activities and its phenolic contents of *Cinnamomum japonicum* Sieb’s extracts (the leaves and branches’s extracts of *Cinnamomum japonicum* Sieb with hot water, 70% EtOH, 100% EtOH, and ultrasonic waves), as in a previous study. It was revealed that the antioxidant activities and phenolic contents were higher in the branches than in the leaves. Additionally, it had the highest antioxidant activity and phenolic compound content when extracted with 70% EtOH (CJB) [23]. Therefore, in this study, the antioxidant activities of fractionated CJB extracts were measured using DPPH and ABTS radical-scavenging assays. In the DPPH and ABTS radical-scavenging assays, the antioxidant activity was highest in CJB3, followed by CJB4, CJB1, CJB2, and CJB5 (Figure 2A). In the TFC assay, the antioxidant activity was highest in CJB3, followed by CJB4, CJB2, CJB5, and CJB1. Additionally, CJB3 had the highest TPC and TFC among the CJB extracts (Figure 2B). High-performance liquid chromatography (HPLC) analysis was performed to identify the CJB3 components. The major component was epicatechin, followed by epigallocatechin gallate and cinnamyl acetate.

The anti-inflammatory efficacy of CJB3, which has high antioxidative activity, in LPS-activated macrophages was investigated. ROS and reactive nitrogen species (RNS) production during phagocytosis in immune cells plays a key role in inflammatory responses [24]. However, excessive ROS accumulation in cells damages macrophages and adjacent tissues, consequently contributing to the pathogenesis of inflammatory diseases [4]. 

The suppressing effect of CJB3 on oxidative stress in LPS-activated cells was investigated first. CJB3 dose-dependently decreased LPS-stimulated ROS and inhibited LPS-induced cellular GSH levels (Figure 4). CJB3 inhibited inflammatory responses like NO production and proinflammatory cytokines (TNF-α and IL-6) (Figure 5 and Figure 6). 

The induction of NO release and proinflammatory cytokines in immune cells is mediated by the activation of inflammatory transcription factors *(NF*-*κB* and AP-1). NF-*κB,* the major inflammatory transcription factor, exists in quiescent cells as homo- or heterodimers bound to the IκB family proteins and is retained in the cytoplasm in an inactive state [25]. However, upon the activation of cells via immune stimulus, IκB is degraded by activated IKK [26]. AP-1 consists of a heterodimer between c-Fos and c-Jun and plays a central role in the transcriptional regulation of inflammatory signaling. Significant upstream proteins in the induction of AP-1 activation are MAPKs (ERK, JNK, and p38) [27]. In this study, the transcription factors (*NF-*κB** and AP-1) that influence CJB3-induced inhibition of inflammatory gene expression were identified. CJB3 suppressed the activation of AP-1 but not that of NF-κB while CJB3 inhibited c-Jun phosphorylation in the lysate cells but not c-Fos phosphorylation. Furthermore, LPS treatment increased the phosphorylation of three types of MAPKs (ERK, JNK, and p38), whereas CJB3 treatment inhibited p38 and JNK phosphorylation (Figure 7). 

In conclusion, the study results indicate that CJB3 prevents LPS-induced oxidative stress and inflammatory responses. In addition, CJB3 exerts its anti-inflammatory effect via the inhibition of p38 and JNK phosphorylation. These results suggest that CJB3 may be a promising candidate for the treatment of inflammatory diseases.

## 4. Material and Methods

### 4.1. Chemical Extracts of Cinnamomum japonicum Sieb Branch

CJ was obtained from Wando-gun, Jeollanam-do, Korea, during the summer of 2020. The fresh CJ branches were dried at 40 °C. The dried branches (100 g) were powdered and extraction was conducted (Figure 1). The extracts were obtained from CJ branches using 70% EtOH for seven days at room temperature (25 ± 2 °C). The extracts were then separated using hexane (CJB1), chloroform (CJB2), ethyl acetate (CJB3), butanol (CJB4), and water (CJB5). All the extracts were vacuum-concentrated (Figure 1).

### 4.2. Antioxidative Activities and Phenolic Contents

#### 4.2.1. DPPH Radical Scavenging Activity

The DPPH radical-scavenging activity was measured using the modified method of Blois [28]. Each concentration sample (200 μL) and 800 μL of 0.5 mM DPPH reagent were mixed, vortexed, and reacted in the dark for 15 min. The absorbance was measured at 517 nm using a Biotek Synergy HT multi-detection microplate reader. Each sample was analyzed three times to obtain an average value and ascorbic acid was used as a positive control. The radical-scavenging activity of each solution was calculated using the following equation and expressed as a percentage: radical-scavenging activity (%) = (Abs_control_ − Abs_sample_)/Abs_control_ × 100, where Abs_control_ is the absorbance of the MeOH control and Abs_sample_ is the absorbance in the presence of the CJB extracts.

#### 4.2.2. ABTS Radical-Scavenging Activity

ABTS radical-scavenging activity was measured as described by Marinova et al. [29]. ABTS (7 mM) was mixed with 2.45 mM potassium persulfate in the same volume and reacted in the dark for 18 h to form ABTS radicals. The ABTS radical solution was diluted with distilled water and the absorbance value at 730 nm was adjusted to 0.90 ± 0.02. Each concentration sample (200 μL) and 1000 μL of ABTS radical solution were mixed, vortexed, and reacted in the dark for 15 min. The absorbance was measured at 730 nm using a Biotek Synergy HT multi-detection microplate reader. The radical-scavenging activity of each solution was calculated using the following equation and expressed as a percentage: radical-scavenging activity (%) = (Abs_control_ − Abs_sample_)/Abs_control_ × 100, where Abs_control_ is the absorbance of the MeOH control and Abs _sample_ is the absorbance in the presence of the CJB extracts.

#### 4.2.3. Total Phenolic and Flavonoid Contents

TPC was measured using the modified Folin–Ciocalteu method [30]: 500 μL of 1 mg/mL CJB extracts, 500 μL of 0.2 M Folin–Ciocalteu’s phenol reagent, and 500 μL of 2% sodium carbonate were mixed, vortexed, and reacted in the dark for 30 min. The absorbance was measured at 750 nm using a Biotek Synergy HT multi-detection microplate reader. The TPC was expressed as mg/g of GAE based on the calibration curve using the following equation: y = 8.4755x + 0.1105, R^2^ = 0.9779, where x is the absorbance and y is the GAE (mg/g).

TFC was measured using a modified Kim et al. method [31]: 500 μL of 1 mg/mL CJ branch extracts, 100 μL of 10% aluminum chloride, 100 μL of 1 M potassium acetate, 1.5 mL of MeOH, and 2.8 mL of distilled water were mixed, vortexed, and reacted in the dark for 40 min. The absorbance was measured at 415 nm using a Biotek Synergy HT multi-detection microplate reader. TFC was expressed as mg/g of QUE based on the calibration curve using the following equation: y = 3.1736x + 0.0397, R^2^ = 0.9998, where x is the absorbance and y is the QUE (mg/g).

### 4.3. High-Performance Liquid Chromatography with Diode-Array Detection (HPLC–DAD) Analysis

The CJB extracts (CJB1-CJB5) were analyzed quantitatively using HPLC with diode-array detection (HPLC-DAD) (SPD-20A, SHIMADZU Co., Japan). Ten standards (epigallocatechin gallate, epicatechin, *ρ*-coumaric acid, coumarin, cinnamyl acetate, cinnamyl alcohol, *trans*-cinnamic acid, cinnamyl aldehyde, eugenol, and quercetin) were selected for the experiment. HPLC analysis conditions were as follows: the column was a Shim-pack GIS-ODS (C18, 4.6 mm × 250 mm × 5.0 μm, Shimadzu Co., Kyoto, Japan), the flow rate was 0.7 mL/min, the temperature was 30 °C, the injection volume was 20 μL, and the ultraviolet (UV) detector wavelength was 280 nm. For the mobile phase, 0.1% acetic acid in water (solvent A) and 0.1% acetic acid in methanol (solvent B) were used. The gradient conditions of the mobile phase were 0 min, B (10%); 0–5 min, B (10%); 5–15 min, B (40%); 15–45 min, B (60%); 45–55 min, B (80%); 55–60 min, B (100%); 60–65 min, B (10%); and 65–70 min, B (10%). The injection volume was 20 μL. All analyzed samples were filtered using a 0.45 µm filter. 

### 4.4. Cell Culture 

Raw 264.7 cells obtained from the ATCC (American Type Culture Collection, Manassas, VA) were maintained in Dulbecco’s Modified Eagle Medium (DMEM) containing 50 units/mL penicillin/streptomycin with 10% fetal bovine serum (FBS) at 37 °C in a humidified 5% CO_2_ atmosphere. LPS (Escherichia coli 055:B5) was purchased from Sigma Chemicals (St. Louis, MO), and CJB3 was extracted from CJB directly by the study research team.

### 4.5. Assay of Nitrite Production

NO production was monitored by measuring nitrite content in the culture medium as previously described [32]. Samples were mixed with Griess reagent (Sigma, St. Louis, MO, USA) and a standard curve was constructed using sodium nitrite (Sigma, St. Louis, MO). The absorbance at 548 nm was measured using an ELISA microplate reader (Spectramax, Molecular Devices) after incubation for 30 min.

### 4.6. Cytotoxicity Assay 

To measure cell viability, cells were plated in 48-well plates and treated with chemicals for 24 h. Viable cells were stained with MTT (0.2 mg/mL, 4 h), as previously described [32]. Next, the media were removed and the formazan crystals produced in the wells were dissolved by adding 200 μL of dimethyl sulfoxide. Absorbance at 540 nm was measured using a microplate reader (Spectramax, Molecular Devices, Sunnyvale, CA, USA). Cell viability was defined relative to the untreated control (viability [% control] = 100 × [absorbance of treated sample]/[absorbance of control]). 

### 4.7. ROS Generation Assay of Bambusae Caulis in Liquamen

DCFH-DA is a cell-permeable, non-fluorescent probe that is cleaved by intracellular esterases and converted into highly fluorescent dichlorofluorescein upon reaction with H_2_O_2_. After 500 µM tert-butyl hydroperoxide (*t*-BHP) treatment with HepG2 cells for 3 h, the cells were stained with 10 µM DCFH-DA for 30 min at 37 °C. H_2_O_2_ generation was determined by measuring dichlorofluorescein using a fluorescence microscope (Zeiss, Germany) or fluorescence microplate reader (Jemini, Molecular Device) at excitation/emission wavelengths of 485/530 nm. 

### 4.8. Immunoblot Analysis

Protein extraction, subcellular fractionation, sodium dodecyl sulfate-polyacrylamide gel electrophoresis (SDS-PAGE), and immunoblot analyses were performed according to previously published procedures [33]. Briefly, the samples were separated using 7.5% gel electrophoresis and electrophoretically transferred to a nitrocellulose paper. The nitrocellulose paper was incubated with the indicated primary antibody and incubated with a horseradish peroxidase-conjugated secondary antibody. Immunoreactive proteins were visualized using electrogenerated chemiluminescence (ECL) chemiluminescence detection (Amersham Biosciences, Buckinghamshire, UK). Equal loading of proteins and the integrity of subcellular fractionation were verified using β-actin immunoblotting. 

Antibodies against iNOS, p65 and IκBα were provided by Santa Cruz Biotechnology (Santa Cruz, CA, USA). Phospho-ERK1/2, ERK1/2, phospho-p38, p38, phospho-JNK1/2, JNK1/2, Lamin A/C and phospho-IκBα were obtained from Cell Signaling Technology (Danvers, MA, USA). Horseradish peroxidase-conjugated goat anti-rabbit, anti-mouse, and anti-goat antibodies were purchased from Invitrogen (Carlsbad, CA, USA). The β-actin antibody was purchased from Sigma Chemicals (St. Louis, MO, USA). 

### 4.9. Statistical Analysis

One-way analysis of variance (ANOVA) was used to assess the statistical significance of the differences among the treatment groups. For each statistically significant treatment effect, the Newman–Keuls test was used for comparisons between multiple group means. The data are expressed as mean ± standard deviation (SD) or SE.

## 5. Conclusions

Overall, the study results indicate that CJB3 prevents LPS-induced oxidative stress and inflammatory responses. Additionally, the anti-inflammatory effect of CJB3 is exerted via the inhibition of p38 and JNK phosphorylation. These results suggest that CJB3 may be a promising candidate for the treatment of inflammatory diseases.

## Figures and Tables

**Table 1:** Total polyphenol content (TPC) and total flavonoid content (TFC) of CJBs.

Sample	DPPH IC_50_ (μg/mL)	ABTS IC_50_ (μg/mL)	TPC	TFC
			GAE mg/g	QUE mg/g
CJB1	643.07 ± 52.19	306.48 ± 2.79	12.39 ± 0.68	1.94 ± 5.80
CJB2	793.32 ± 15.37	168.45 ± 1.15	19.21 ± 4.71	16.37 ± 1.04
CJB3	144.72 ± 4.58	35.08 ± 0.06	50.65 ± 1.64	143.28 ± 6.34
CJB4	243.02 ± 7.62	67.10 ± 0.71	30.60 ± 0.72	62.08 ± 1.04
CJB5	827.06 ± 16.51	313.18 ± 2.28	10.92 ± 0.13	4.94 ± 1.80
Catechin	73.63 ± 1.86	17.18 ± 0.87	-	-

## Data Availability

The data presented in this study are available in this article.

## Supplementary Materials

The following supporting information can be downloaded at: https://www.mdpi.com/article/10.3390/molecules28041974/s1, Figure S1. Quantitative analysis of polyphenols using HPLC MS/MS; Table S1. The conditions of HPLC MS/MS; Table S2. The contents of polyphenols of the extracts.

## Sample Availability

Samples of the compounds used in this study are not available from the authors.
