# Supplementary material for: Cinnamomum japonicum Siebold Branch Extracts Attenuate NO and ROS Production via the Inhibition of p38 and JNK Phosphorylation
Source: Molecules. 2023 Feb 19;28(4):1974. doi: 10.3390/molecules28041974 (PMC9960860; doi:10.3390/molecules28041974)
Supplement: Supplementary file 1 [file molecules-28-01974-s001.zip › molecules-2162865-supplementary.pdf]

Supplementary Materials. Quantitative analysis of polyphenols using HPLC MS/MS

Table S1. The conditions of HPLC MS/MS

		Negative	Positive
Standard		Epigallocatechin gallate, p-Coumaric acid, Quercetin	Epicatechin, t-Cinnamic acid, Coumarin, Cinnamylaldehyde, Eugenol
MS	Condition	Turbo Ion Spray, temperature 500℃, MRM scan type spray voltage -4500V, CG 20, GS1 50, GS2 50.	Turbo Ion Spray, temperature 500℃, Spray voltage 5500V, CG 20, GS1 50, GS2 50
LC	Column	Gemini 3 ㎛, C18 110A 50 ㎜*2.0 ㎜<br>Gemini C18(4.0 ㎜ × 2.0 ㎜) guard cartridge	Gemini 3 ㎛, C18 110A 50 ㎜*2.0 ㎜<br>Gemini C18(4.0 ㎜ × 2.0 ㎜) guard cartridge
	Temperature	40℃	40℃
	Autosampler	15℃	15℃
	Gradient time	10% B start, 0.5min-40% B, 1.0min-85% B, 3min-85% B, 3.1min-10% B, 6min stop	10% B start, 0.5min-40% B, 1.0min-85% B, 3min-85% B, 3.1min-10% B, 6min stop
	Flow rate	0.3 mL/min	0.3 mL/min

Table S2. The contents of polyphenols of the extracts

No.	Standard	Calculated Concentration CJB3 (μg/g)
1	Epigallocatechin gallate	3.13
2	Epicatechin	0.48
3	p-Coumaric acid	31.11
4	Coumarin	0.02
5	Cinnamyl acetate	-
6	Cinnamyl alcohol	-
7	trans-Cinnamic acid	3.96
8	Cinnamyl aldehyde	-
9	Eugenol	0.40
10	Quercetin	98.82
	Total	137.92
